# Gastric atrophy prevent from incidence of duodenal tumor?

**DOI:** 10.3164/jcbn.16-74

**Published:** 2017-05-01

**Authors:** Takashi Kawai, Mami Takeuchi, Masakatsu Fukuzawa

**Affiliations:** 1 Gastroenterological Endoscopy, Tokyo Medical University, 6-7-1, Nishisinjuku, Shinjuku-ku, Tokyo, 160-0023, Japan; 2 Gastroenterology and Hepatology, Tokyo Medical University, 6-7-1, Nishisinjuku, Shinjuku-ku, Tokyo, 160-0023, Japan

Recently there has been a noticeable increase in the number of papers describing diagnosis and treatment of non-ampullary duodenal epithelial tumors (NADET). The authors investigated the link between NADET and gastric atrophy in 61 endoscopic treatments carried out at this hospital from 2,000 onwards. The average age within the sample population was 61.8 ± 11.4 years, with a male to female ratio of 47:14. There were 48 cases of tubular adenoma and 13 of cancer. Some 82% of endoscopic gastric atrophy were classed as the close types (C) under the Kimura-Takemoto classification system for endoscopic gastric atrophies (C-1 = 41 cases, C-2 = 5 cases, C-3 = 4 cases); on the other hand open types (O) were O-1 = 1 case, O-2 = 10 cases, O-3 = 0 cases. While a link between gastric cancer and advanced gastric atrophy has been reported previously, the results suggest a low incidence of gastric atrophy in NADET. Further research is needed into potential correlations between NADET and physiological phenomena such as gastric atrophy, gastric acid secretion and bile activity.

